# PReS-FINAL-2172: Efficacy of corticosteroids and intravenous cyclophosphamide for patients with juvenile systemic sclerosis

**DOI:** 10.1186/1546-0096-11-S2-P184

**Published:** 2013-12-05

**Authors:** M Kikuchi, T Nozawa, T Kanetaka, K Nishimura, R Hara, K Yamazaki, T Sato, N Sakurai, S Yokota

**Affiliations:** 1 Department of pediatrics, Yokohama city univercity school of medicine, Yokohama, Japan

## Introduction

Systemic sclerosis (ssc) is a rare multisystemic disease characterized by inflammation, vascular abnormalities, and fibrosis that affects the skin and various internal organs. Juvenile ssc accounts for fewer than 10% of all adults with ssc. Regarding effective treatment there were no specific pediatric data available, and the long-term efficacy of treatment for children with ssc has not been investigated.

## Objectives

To evaluate efficacy of treatment in juvenile ssc and to extract factors related to poor prognosis.

## Methods

Ten patients (4 boys and 6 girls) with ssc were included. All of them were diagnosed as a diffuse type ssc based on clinical manifestations (Raynaud's phenomenon, skin induration and/or internal organ involvements), serological findings, and imaging assessments of internal organ damages. The responses to treatment during their clinical courses were assessed by Total Skin Score (TSS) and internal organ damages using imaging assessment modalities.

## Results

Average onset age was 10.3 years and average duration until diagnosis was 22 months (range 2-74 months). Average observation period was 41 months. Nine out of 10 patients had antinuclear antibodies, and anti-Scl-70 antibody was positive in 4. Nine patients were revealed upper gastrointestinal dysfunction, 2 were shown interstitial lung disease, the other 2 were detected pulmonary hypertension, and 1 had arrhythmia. All of the patients were treated primarily with corticosteroids, followed by intravenous cyclophosphamide (IVCY) (12 months course) as induction therapy. Most of the patients received oral prednisolone and other immunosuppressants such as azathioprine, methotrexate, and mycophenolate mofetil as maintenance therapy. Six out of 10 patients were shown improvement of TSS, and 2 patients with pulmonary hypertension had remarkable improvement after 2-year IVCY therapy. In 4 out of these 6 patients both TSS and internal organ damages were improved, and the other 2 patients ameliorated in TSS but not in internal organ damages. TSS of 3 patients with the positive anti-Scl-70 antibody was unchanged or increased suggesting anti-Scl-70 antibody may be one of poor prognostic factors. Other 2 patients with the positive anti-Scl-70 antibody were complicated with interstitial lung disease, and they were refractory to the treatment suggesting again the anti-Scl 70 antibody may be one of the poor prognostic factors. Additionally, these 2 patients received incomplete IVCY therapy due to anaphylactic reaction to IVCY or patient's refusal. One female with the positive anti-Scl-70 antibody died due to acute heart failure with no appropriate therapy in a regional hospital, and then after being transferred to our hospital methylprednisolone pulses and IVCY was successfully administered for TSS improvement during 3 months, but lung fibrosis and arrhythmia were progressed.

## Conclusion

The earlier diagnosis and induction of corticosteroids and IVCY therapies will be indispensable for the prevention of fatal organ involvement in juvenile ssc. Incomplete immunosuppressive therapies and the positive detection of the anti-Scl-70 antibody may be poor prognostic factors.

## Disclosure of interest

None declared.

